# Tolerant/Persister Cancer Cells and the Path to Resistance to Targeted Therapy

**DOI:** 10.3390/cells9122601

**Published:** 2020-12-04

**Authors:** Mirna Swayden, Houssein Chhouri, Youssef Anouar, Luca Grumolato

**Affiliations:** 1 Laboratoire Différenciation et Communication Neuronale et Neuroendocrine, UNIROUEN, INSERM, Normandie Université, 76183 Rouen, France; mirna.swayden@univ-rouen.fr (M.S.); houssein.chhouri@univ-rouen.fr (H.C.); youssef.anouar@univ-rouen.fr (Y.A.); 2 Institute for Research and Innovation in Biomedicine, 76183 Rouen, France

**Keywords:** targeted therapy, drug resistance, tolerant and persister cells, intratumor heterogeneity, cell signaling, melanoma, lung cancer, BRAF, EGFR

## Abstract

The capacity of cancer to adapt to treatment and evolve is a major limitation for targeted therapies. While the role of new acquired mutations is well-established, recent findings indicate that resistance can also arise from subpopulations of tolerant/persister cells that survive in the presence of the treatment. Different processes contribute to the emergence of these cells, including pathway rebound through the release of negative feedback loops, transcriptional rewiring mediated by chromatin remodeling and autocrine/paracrine communication among tumor cells and within the tumor microenvironment. In this review, we discuss the non-genetic mechanisms that eventually result in cancer resistance to targeted therapies, with a special focus on those involving changes in gene expression.

## 1. Introduction

Cancer is the second leading cause of death worldwide [1]. The current treatment strategies for this disease include surgery, chemotherapy and radiotherapy, all of which have clinical limitations. Radiations can induce damage of surrounding tissues leading to poor wound healing [2], while surgery is often not suitable for metastatic patients, and chemotherapy tends to target highly proliferating normal cells, leading to systemic toxicities with adverse side effects [3]. In recent years, new immunotherapy approaches have shown remarkable response rates for the treatment of certain types of cancer, but their efficacy in a wider range of tumors remains to be demonstrated. Another therapeutic strategy relies on the specific targeting of genes and/or signaling pathway components that drive tumorigenesis and tumor progression, thus limiting side effects, while providing high response rates. In most cases, these anti-tumor agents are represented by small molecule kinase inhibitors or monoclonal antibodies that target pathways involved in the proliferation and survival of tumor cells, such as, for example, those triggered by receptor tyrosine kinases (RTK), including the epidermal growth factor receptor (EGFR) and HER2, or by downstream effectors, including the kinases BRAF and ABL. In the U.S., the Food and Drug Administration has already approved more than 50 small molecule kinase inhibitors [4] and several monoclonal antibodies [5] for targeted therapy against several types of tumor. Unfortunately, despite an often-remarkable initial response, the acquisition of resistance and patient relapse are observed in most cases.

**Figure 1:**
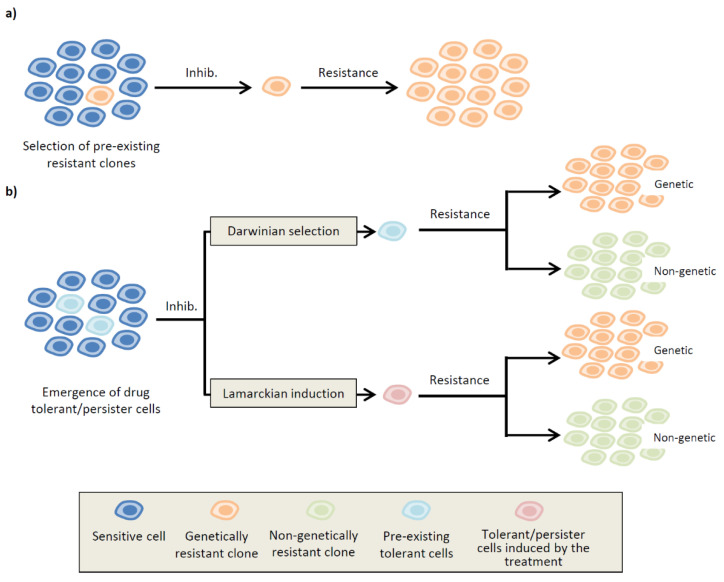
Evolutionary model for the development of drug resistance in cancer cells. (**a**) Rare pre-existing clones containing resistance mutations (orange) are selected in response to treatment with a specific inhibitor (Inhib.). (**b**) Alternatively, resistance may arise from tolerant/persister cells capable of surviving in the presence of the inhibitor (light blue and red) and evolve over time to acquire genetic (orange) or non-genetic resistance (green). These cells are selected because of some intrinsic properties (light blue) through non-genetic Darwinian selection, or they can originate more randomly (red), as a direct effect of the inhibitor (Lamarckian induction).

A tumor is a mosaic entity composed of distinct cell subpopulations with a potentially different genetic and/or epigenetic profile. The concept of intratumoral heterogeneity has important implications for cancer therapy, since individual clones can respond differently to treatment. Indeed, studies have indicated that patients with higher intratumoral heterogeneity have poorer prognosis with a worse clinical outcome [6,7]. It has been shown that, in some cases, the treatment can provoke the emergence of pre-existing clones containing resistance mutations, resulting in tumor relapse (Figure 1a) [8,9]. More frequently, some cells escape cell death in the presence of the drug, thus enabling the maintenance of a residual tumor mass. Notably, these surviving cells can behave in distinct patterns: for simplicity, here, we refer to cells displaying lower drug sensitivity as tolerant cells, and cells entering a state of dormancy to evade drug-induced apoptosis as persister cells [10,11,12]. It has been proposed that tolerant/persister cells can functionally lead to the emergence of resistant clones through the acquisition of de novo mutations [9,13]. However, the situation is more complex, as non-genetic mechanisms of resistance have been demonstrated in several cancer types [12,14,15,16,17,18]. These observations imply that tolerant/persister cells can give rise to resistant clones through new specific mutations or by selecting a particular cell state that enables growth in the presence of the drug (Figure 1b).

## 2. Reactivation of the Pathway Targeted by the Drug

**Figure 2:**
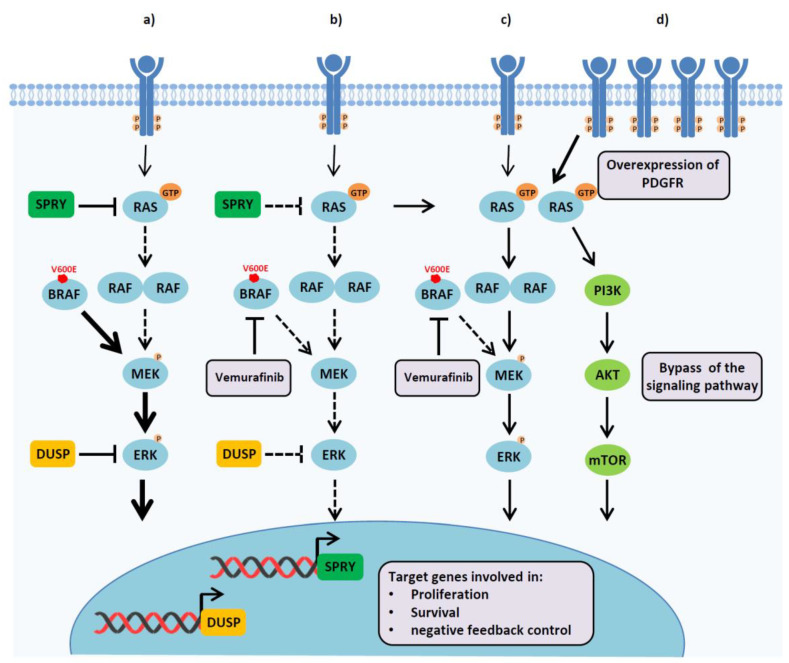
Reactivation of the mitogen-activated protein kinase (MAPK) pathway resulting from the relief of negative feedback loops. (**a**) In melanoma cells containing the BRAF-V600E mutation, MAPK signaling is highly active and leads to enhanced expression of several genes promoting cell proliferation and survival, but also of other genes involved in negative feedback loops, including DUSP and SPRY. (**b**,**c**) Pharmacological inhibition of the MAPK pathway with the BRAF inhibitor vemurafenib provokes downregulation of DUSP and SPRY (**b**), thus relieving the negative feedback loop (**c**). In these conditions, some level of signaling can be reactivated independently of the BRAF mutation. (**d**) A similar adaptive response to BRAF inhibition relies on the overexpression of PDGFR, resulting in an upstream activation of the pathway that bypasses mutant BRAF.

In normal conditions, signaling pathways are tightly regulated by complex feedback mechanisms, which often involve gene expression changes. Aberrant activation of these pathways through oncogenic mutations is a common event in cancer, which can drive tumor formation and progression. While targeted therapies can effectively inhibit the signaling, in some cases they can also affect negative feedback loops, resulting in reactivation of the pathway. As a typical example, in physiological conditions, excessive stimulation of Ras/mitogen-activated protein kinase (MAPK) signaling can be prevented by extracellular signal-regulated kinase (ERK)-dependent induction of negative regulators, including Sprouty (Spry) and dual specificity phosphatases (DUSP). In melanomas, mutant BRAF aberrantly activates the pathway downstream of Ras, thus bypassing this feedback loop. Inhibitors, such as vemurafenib, block ERK activation induced by BRAF monomers, resulting in Spry and DUSP downregulation. In these conditions, a rebound of the MAPK pathway can be observed due to an upstream, ligand-dependent activation of the signaling, which does not involve mutant BRAF (Figure 2a–c) [19]. A similar adaptive mechanism of resistance in vemurafenib-treated melanomas relies on drug-induced overexpression of the platelet-derived growth factor receptor (PDGFR), which, as the other members of RTK family, is an upstream inducer of the Ras-MAPK signaling (Figure 2d) [20]. Feedback reactivation of RTKs induced by treatment can also occur through post-translational mechanisms, as it has been shown in melanoma and breast cancer cells, where MAPK inhibition can reduce the proteolytic shedding of AXL, MET and HER4, thus increasing the levels of these receptors at the cell surface [21].

The clinical implications of this type of regulatory loops often depend on the cell context. As an example, while very effective to treat BRAF mutant melanomas, BRAF inhibitors showed poor responses in colon cancers harboring the same oncogenic mutation. This paradox could mechanistically be explained by the existence of a negative feedback loop that causes EGFR inhibition when the MAPK pathway is strongly activated. This mechanism has limited consequences in melanoma cells, which express low levels of EGFR. In colon cancer, the expression levels of this receptor are higher, and BRAF inhibition causes a relief of the regulatory loop, resulting in pathway rebound induced by upstream EGFR activation. As a consequence, it was shown that concurrent inhibition of BRAF and EGFR strongly reduces the growth of BRAF mutant colon cancer cells, both in vitro and in vivo [22,23]. These types of strategies based on vertical inhibition, where multiple components of the same pathway are targeted simultaneously, are used in the clinic for certain tumors, including melanoma [24], to delay the emergence of drug resistance.

While providing a means to escape targeted therapy, adaptive pathway reactivation also exposes cancer cells to a new form of vulnerability that could be conceivably exploited to treat patients. Indeed, upon drug withdrawal, sudden release of oncogene inhibition can provoke, in some cases, an overactivation of the pathway that is toxic for the cells, potentially resulting in the regression of resistant tumors [25,26,27]. As an example of this type of cancer cell drug addiction, it has been shown in melanoma cells that, upon cessation of treatment with BRAF and MEK inhibitors, ERK2-mediated induction of the activator protein 1 (AP1) transcription factors JUNB and FRA1 can trigger a switch in gene expression, including downregulation of B-cell lymphoma 2 (BCL2), which provokes apoptosis [28].

To explore the therapeutic potential of this type of vulnerability in a preclinical setting, Das Thakur et al. reported that intermittent dosing of vemurafenib based on a 4-week on and a 2-week off schedule enhances drug sensitivity and prolongs survival in a mouse model [26]. This type of approach based on “drug holiday” cycles is expected to delay the emergence of resistant clones, in line with other studies showing that continuous dosing favors the clonal expansion of resistant cells [29]. This strategy is an example of adaptive therapy, which postulates that treatment should be constantly modulated to reach a stable population of sensitive cells capable of growing over the less fit populations of resistant cells [30]. While this concept is supported by preclinical studies [26,28,31] and mathematical modeling [32], its applications in the clinic may prove to be more difficult: in a recent randomized phase two clinical trial in melanoma patients treated with a combination of BRAF and MEK inhibitors, intermittent dosing did not improve progression-free and overall survival [33].

## 3. Activation of Other Pathways and Emergence of Tolerant/Persister Cells

In addition to pathway rebound mediated by the release of negative feedback loops, other mechanisms can negatively affect the response of cancer cells to targeted therapy. In melanoma, BRAF and MEK inhibitors can induce an upregulation of the transcription factor PAX3, which in turn stimulates the expression of the microphthalmia-associated transcription factor (MITF), ultimately promoting cell survival [34]. Other studies suggest that MITF may also mediate a metabolic rewiring in cancer cells by increasing the levels of the mitochondrial master regulator PGC1α, resulting in a switch from glycolysis to oxidative phosphorylation [35]. This change in energy metabolism could also improve the detoxification capacities of cancer cells against reactive oxygen species, thus facilitating survival under oxidative stress conditions [36]. Indeed, other studies showed that tolerant/persister cells are particularly vulnerable to strategies designed to target antioxidant mechanisms, such as those involving inhibitors of the glutathione peroxidase 4, which were shown to specifically induce ferroptosis [37].

As another example of therapy-induced activation of alternative pathways, a recent study reported that non-small-cell lung cancer (NSCLC) cells tolerant to EGFR inhibitors display an increased activity of Aurora kinase A (AURKA), mediated by an upregulation of the TPX2 protein. The authors found that AURKA enables survival of tolerant cells by phosphorylating the pro-apoptotic factor BIM, resulting in its proteasomal degradation. Of note, high levels of TPX2 and AURKA can also provoke mitotic errors and lead to chromosomal instability, which may enhance intratumor heterogeneity and favor the emergence of resistance mutations [14].

A different mechanism enabling survival of both melanoma and NSCLC cells in the presence of targeted therapy involves upregulation of the RTK AXL and the subsequent induction of an epithelial-to-mesenchymal transition (EMT) [38,39]. In particular, high levels of AXL in melanoma are associated with a transcriptional reprogramming that induces a switch from a proliferative to an invasive phenotype, intrinsically less sensitive to inhibition of the BRAF/MAPK pathway and characterized by low levels of MITF expression [39]. It was shown that small subpopulations of AXL-positive cells are present in untreated melanoma samples, as well as patient-derived xenografts (PDXs), implying that they can be positively selected in the presence of BRAF/MEK inhibitors. As an alternative mechanism, the same study also reported acute transcriptional induction of AXL by inhibition of the MAPK pathway. To delay drug resistance and prolong patient response, the authors proposed a therapeutic strategy based on specific targeting of tolerant cells using an anti-AXL antibody-drug conjugate [40]. This study highlights an important question in the field: how do tolerant/persister cells originate? As in the case of AXL-positive cells in untreated melanomas, pre-existing subpopulations can emerge through non-genetic Darwinian selection because of particular intrinsic properties that favor growth in the presence of the drug. Alternatively, and according to a different model, the treatment could induce gene expression changes that provoke a cell state switch to a tolerant/persister phenotype. This process, defined as Lamarckian induction [41,42] (Figure 1b), implies that tolerant/persister cells arise more randomly, as a consequence of a cell fate decision possibly influenced, at least in part, by stochastic fluctuations of gene expression [43,44] or a particular phase of the cell cycle, as it has been shown for embryonic stem cells [45]. A combination of these two models is also conceivable, where certain subpopulations are selected based on a particular pattern of gene expression, followed by additional changes induced by the drug. An example of this dual mechanism has been recently described for melanoma, with the identification of rare cell subpopulations displaying a particular transcriptional profile, characterized by high levels of resistance markers. These cells are primed to survive in the presence of the drug because of a transient tolerant state, which can then be converted by the treatment to a more stable resistant phenotype through epigenetic reprogramming, possibly engendered by inhibition of SOX10-mediated differentiation and induction of AP1 and TEAD transcription factors [15]. Using an elegant strategy based on Luria and Delbrück fluctuation analysis [46], in a follow-up paper the same authors showed that changes in the expression of certain genes across the genome can occur at the single cell level and persist for several divisions in different types of cancer. Such biological memory relies on heritable expression programs that single cells use to adapt to environmental conditions, including therapeutic intervention [47].

While some cancer cells can be transcriptionally primed to behave as tolerant, it has been shown that other cells can survive in the presence of the treatment by entering a dormancy state. Cellular barcoding experiments in NSCLC lines suggest that this process is randomly induced by EGFR/MEK inhibitors, which enable some cells to adopt a reversible senescence program and escape death [11]. From this residual pool of cells, resistant populations can arise through either genetic or non-genetic events. Mechanistically, NSCLC quiescent cells display upregulation of the YAP/TEAD pathway, which represses the pro-apoptotic factor BMF through interaction with the EMT transcription factor SLUG and thereby blocks apoptosis [11]. Consistent with this model, pharmacological inhibition of YAP/TEAD can deplete the reservoir of persister cells, resulting in an enhanced NSCLC response to EGFR/MEK [11] and anaplastic lymphoma kinase (ALK) [48] inhibitors. Of note, activation of the YAP/TEAD pathway can also compensate the loss of another dominant oncogene, KRAS, and promote survival in the absence of MAPK activation in different types of addicted tumor cells, including those derived from colon, lung and pancreatic cancer [49,50].

The recent advances in single-cell transcriptomics provide a new means to investigate how tumors acquire resistance to targeted therapy. In a recent study using melanoma PDXs, the group of J.C. Marine revealed that, during the minimal residual disease phase induced by treatment with BRAF/MEK inhibitors, distinct drug tolerant transcriptional states coexist. These different subpopulations showed spatial heterogeneity within tumors, as well as a marked variability between samples derived from distinct patients. In addition to the previously described AXL-positive and MITF-positive cells, the authors also identified a distinct population displaying a neural crest stem cell (NCSC) gene signature. Computational analysis revealed a gene regulatory network in which the retinoid X receptor-γ (RXRG) constitutes a critical node. To confirm the role of this transcription factor in the induction of a tolerant phenotype, they co-treated melanoma PDXs with a combination of BRAF/MEK inhibitors and an RXR antagonist, which resulted in a decrease in the levels of NSCLC markers and a delay in the acquisition of resistance [51]. The identification of a population of tolerant cancer cells displaying a NCSC transcriptional profile is consistent with the general notion that cancer stem cells respond poorly to treatment. The mechanistic basis for this low sensitivity could be related to the fact that cancer stem cells often express high levels of transporters that can mediate the efflux of drugs. Moreover, cancer stem cells can rely on alternative pathways for their maintenance, making them less addicted than other cancer cells to the driving oncogene targeted by the therapy [52].

As described above, targeted therapies can provoke, through different mechanisms, phenotypic changes that are ultimately responsible for tumor relapse. An extreme example of this type of plasticity is represented by the histologic transformation toward a small cell neuroendocrine (SCN) phenotype observed in NSCLC and prostate cancer treated with EGFR inhibitors [53,54] or hormone therapy [55], respectively. While genetic events are probably necessary for this type of trans-differentiation, they are not sufficient and they are accompanied by a transcriptional reprogramming induced by non-genetic mechanisms [56,57]. In a recent study, a SCN gene expression signature based on epigenomic modifications was identified across different types of cancer and associated with resistance to therapy. Of note, solid cancer cells displaying the SCN signature showed drug vulnerabilities similar to those observed in hematological malignancies, which could provide a potential strategy for therapy [58].

## 4. Chromatin Remodeling Can Modulate Gene Expression and Promote Drug Tolerance and Resistance

Transcriptional adaptation during the emergence of non-genetic resistance can be mediated by changes in key epigenetic drivers. Histone modifying enzymes regulate chromatin architecture and participate in the activation or repression of gene expression. Upon drug exposure, the activity of these enzymes can be altered, thus yielding a transcriptional profile that favors cell survival. Recently, SETD5 was identified as a chromatin-based master mediator of resistance to MEK inhibitors in pancreatic cancer. Upon acquisition of resistance to these inhibitors, it was shown that pancreatic cell lines and PDXs overexpress SETD5, while inactivation of this gene by CRISPR/Cas9 restores drug response. Mechanistically, the authors found that SETD5 lacks intrinsic histone lysine methyltransferase activity, but it can form a co-repressor complex with the histone deacetylase 3 (HDAC3) and the methyltransferase G9a, which coordinates deacetylation and methylation of histone H3 lysine K9 (H3K9). The gene expression program mediated by this complex, including downregulation of genes involved in the cytochrome P450 pathway and glutathione metabolism, can promote pancreatic cancer resistance to MEK inhibitors. Indeed, combination of MEK inhibitors with compounds targeting G9a and HDAC3 strongly delayed the growth of pancreatic PDXs with minimal secondary effects, suggesting that a similar strategy could be used in the clinic to prolong therapeutic response in patients [59].

A major feature of enhancer elements is their ability to form binding scaffolds for activating or repressing transcription factors. Enhancer remodeling is a new mode of adaptive transcriptional plasticity in response to treatment, where cells use alternative enhancers to drive the expression of a subset of survival genes associated with the resistant state. A recent study reported that, in ALK mutant, MYCN amplified neuroblastomas, resistance to ALK inhibitors is accompanied by a decrease in MYCN expression and upregulation of the brother of the regulator of imprinted sites (BORIS) protein. BORIS is a CCCTC-binding factor that can anchor DNA loops to form structural domains within the genome that affect gene expression. In ALK resistant neuroblastoma cells, BORIS can induce the formation of new super-enhancers that drive the expression of a group of pro-neural transcription factors, including neurogenic differentiation 1 and 2, SRY-box 2 and 9 and Achaete-scute complex homolog 2, promoting a phenotypic switch in resistant cells [60]. Mechanisms involved in enhancer switching during the acquisition of resistance can represent a potential target to restore drug sensitivity. In an acute myeloid leukemia murine model of non-genetic resistance to inhibitors of the bromodomain and extra-terminal (BET) family of proteins, Bell *et al.* found that targeting of the lysine-specific histone demethylase 1 (LSD1) can induce the formation of new enhancers and restore sensitivity to the drug. Mechanistically, LSD1 inhibition allows the binding of the transcription factor Pu.1 and its cofactor interferon regulatory factor 8 to generate new enhancers. These new enhancers regulate the expression of important survival genes through recruitment of the transcriptional co-activator bromodomain-containing protein 4 (BRD4), thus re-sensitizing the cells to BET inhibitors [16]. Of note, resistance to NOTCH targeting using γ-secretase inhibitors in T cell acute lymphoblastic leukemia (T-ALL) is accompanied by increased binding of BRD4 to enhancers located in the proximity of genes encoding important factors involved in cell proliferation and survival, such as MYC and BCL2. Consistent with these findings, a combination of BET and γ-secretase inhibitors synergistically delayed the progression of human primary T-ALL cells upon transplantation in immunodeficient mice [61].

The reversibility of the drug tolerant phenotype strongly suggests the implication of an epigenetic mechanism. Indeed, it has been shown that the emergence of tolerant NSCLC cells could be prevented by co-treatment with HDAC inhibitors [10]. In a follow-up study, the same authors reported that these cells display a repressed chromatin status characterized by H3K9 and H3K27 methylation of genomic loci containing transposable elements, such as long interspersed repeat element 1 (LINE-1). Treatment with HDAC inhibitors de-repressed LINE-1 expression and induced cell death in the subpopulation of tolerant cells, while this effect was partially blocked by siRNA mediated downregulation of LINE-1. These data lead the authors to speculate that repression of transposable elements may provide the cells with a reversible genome protective mechanism ensuring survival during drug treatment [62].

## 5. Autocrine/Paracrine Signaling can Participate in the Resistance of Cancer Cells to Therapy

It has been shown that targeted therapy can affect the expression by tumor cells of certain secreted factors, which can then signal in an autocrine/paracrine manner to modulate different cancer properties, including drug sensitivity. For example, MAPK inhibition in different types of cancer cells addicted to mutant KRAS or RTKs, such as EGFR (NSCLC), HER2 (breast cancer) or ALK (neuroblastoma), can provoke an increase in the secretion of fibroblast growth factor and interleukin-6. Through autocrine activation of their cognate receptors, these factors then stimulate the STAT3 pathway, thus favoring cell survival in the presence of treatment. Consistent with this model, the study reported that pharmacological targeting of this autocrine loop could prevent resistance to MAPK inhibition and promote tumor regression [63].

As described in previous sections, tumor relapse can be caused by the emergence, during treatment, of a pre-existing subpopulation of genetically resistant cells. Using a mixing experiment approach based on fluorescently labeled cells, the laboratory of J. Massagué found that targeted therapy against EGFR, ALK and BRAF could enhance the in vivo growth of resistant cells when interspersed with drug sensitive cells. Mechanistically, the authors demonstrated that treatment can induce downregulation of the AP1 transcription factor FRA1 in sensitive cells, resulting in the release of different secreted proteins, including EGF, insulin-like growth factor 1, angiopoietin-like 7 and platelet-derived growth factor D. These ligands can then signal in a paracrine manner to resistant cells, inducing the activation of the AKT pathway and promoting growth [64].

The tumor microenvironment (TME) consists of an extracellular matrix and different types of normal cells, including cancer associated fibroblasts (CAFs), myofibroblasts, inflammatory cells, endothelial cells, pericytes and dendritic cells. Interactions between tumor cells and the TME are highly dynamic and involve various cytokines, chemokines and growth factors that can affect tumor progression, as well as response to therapy [65]. Among their pleiotropic effects on tumor cells, CAFs participate in drug resistance through different mechanisms [66]. Using intravital imaging to detect ERK activation in melanoma cells, Hirata et al. showed that vemurafenib can activate and remodel CAFs to generate an extracellular matrix rich in fibronectin and collagen, which can stimulate β1-integrin/focal adhesion kinase/SRC signaling in melanoma cells, resulting in MAPK reactivation. By sheltering cancer cells from BRAF inhibition, this study revealed that CAFs provide a safe haven favoring the formation of resistant clones [67].

CAFs can also secrete growth factors capable of interfering with cancer response to therapy. For example, it was shown that co-culture of prostate cancer cells with CAFs could promote survival in the presence of antiandrogens. The authors identified neuregulin 1 (NRG1) as a factor secreted by the stroma during hormone therapy that can signal to the HER3 receptor expressed by tumor cells. Activated HER3 then enhances the expression of a subset of canonical target genes of the androgen receptor, thus circumventing the effects of therapy. This mechanism was confirmed in different preclinical models, where inhibition of the NRG1/HER3 axis could prevent prostate cancer resistance to antiandrogens [68]. Similar co-culture experiments indicated that CAFs can also promote survival of breast cancer cells upon HER2 inhibition, by inducing an increase in the expression of different antiapoptotic proteins, as well as the activation of the AKT/mTOR pathway [69]. As another example of paracrine signaling between CAFs and tumor cells, the hepatocyte growth factor (HGF) is secreted by the stroma upon treatment with BRAF or EGFR inhibitors, conferring resistance to melanoma [70] and NSCLC cells [71], respectively. Recent studies revealed that RTK inhibitors provoke a metabolic shift toward increased glycolysis in addicted cancer cells. The high levels of lactate produced by tumor cells can then induce an upregulation of HGF secretion from CAFs, with a mechanism dependent on the NF-κB pathway. This growth factor can then bind to its receptor MET expressed by tumor cells, promoting resistance to RTK inhibitors [72]. On the other hand, the communication between the tumor and the surrounding cells can be affected by metabolic changes occurring in the TME. For example, it has recently been shown that aging fibroblasts secrete high levels of different lipids, including ceramides, which can promote melanoma resistance to BRAF and MEK inhibitors [73]. Novel strategies to investigate cancer metabolism at a regional [74] and single cell [75] resolution suggest that the TME can also play another, more indirect role in promoting drug resistance, by generating an intratumor metabolic heterogeneity [76] that can favor the emergence of tolerant/persister cells during treatment.

In addition to fibroblasts, paracrine signaling between tumor and inflammatory cells within the TME niche can also enable oncogenic pathway reactivation and participate in tumor progression. In melanoma, paradoxical activation of the MAPK pathway induced by BRAF inhibitors in macrophages results in upregulation and secretion of the vascular endothelial growth factor, which in turn restores MAPK signaling and promotes resistance in tumor cells [77]. It has also been shown that MEK and BRAF inhibitors can increase the number of macrophages, as well as their expression of tumor necrosis factor-α (TNF-α), mediating upregulation of MITF expression in melanoma cells through NF-κB activation. Consistent with these findings, inhibition of TNF-α signaling using IκB kinase inhibitors exerted a synergistic effect with MEK inhibitors to prevent resistance. Of note, the authors also found that accumulation of macrophages in the TME during treatment could function as a predictor of early relapse in patients [78].

## 6. Conclusions

Acquired resistance is a major drawback of targeted therapies. While mutations unquestionably play a major role in this process, recent findings have demonstrated the important contribution of non-genetic mechanisms in several cancer types. As discussed in this review, changes in the expression of certain genes, specific epigenetic states and paracrine/autocrine cell communication can all participate in the initial survival of tolerant/persister cells in the presence of the treatment, as well as in the transition toward a resistant phenotype. Further advances in single cell analysis and lineage tracing should provide a better understanding of the plasticity and reprogramming capacity of the different subpopulations that constitute a tumor. With this knowledge, new therapeutic strategies could be developed to prevent the adaptive mechanisms responsible for both genetic and non-genetic resistance, thus improving the clinical outcome for cancer patients.
